# Deaf moths employ acoustic Müllerian mimicry against bats using wingbeat-powered tymbals

**DOI:** 10.1038/s41598-018-37812-z

**Published:** 2019-02-05

**Authors:** Liam J. O’Reilly, David J. L. Agassiz, Thomas R. Neil, Marc W. Holderied

**Affiliations:** 1 School of Biological Sciences, University of Bristol, Bristol, UK; 2 Department of Life Sciences, Insect Division, Natural History Museum, London, UK

## Abstract

Emitting ultrasound upon hearing an attacking bat is an effective defence strategy used by several moth taxa. Here we reveal how *Yponomeuta* moths acquire sophisticated acoustic protection despite being deaf themselves and hence unable to respond to bat attacks. Instead, flying *Yponomeuta* produce bursts of ultrasonic clicks perpetually; a striated patch in their hind wing clicks as the beating wing rotates and bends. This wing structure is strikingly similar to the thorax tymbals with which arctiine moths produce their anti-bat sounds. And indeed, *Yponomeuta* sounds closely mimic such arctiine signals, revealing convergence in form and function. Because both moth taxa contain noxious compounds, we conclude they are mutual Müllerian acoustic mimics. *Yponomeuta*’s perpetual clicking would however also attract bat predators. In response, their click amplitude is reduced and affords acoustic protection just as far as required, matching the distance over which bat biosonar would pick up *Yponomeuta* echoes anyway – advanced acoustic defences for a deaf moth.

## Introduction

Bats and moths have been involved in a 65-million-year evolutionary arms race since the advent of biosonar in Chiroptera^1^. As a result, moths have evolved a plethora of defences against their chiropteran adversaries. In addition to hearing structures tuned to the echolocation frequencies of sympatric bats^2,3^ providing an early warning system and allowing time for evasive manoeuvres, sound production as a defence against bats has evolved independently in at least three moth families^4–6^. Many bat species detect and localise prey by the sounds they generate^7,8^, so sound production is only adaptive when it creates protection with the sounds startling attacking bats, warning them of a (chemical) defence or jamming their biosonar^9–11^. These sounds’ acoustic properties such as duty cycle and number of clicks may be used to classify them by function^1,4^.

In addition to the anti-bat sounds of some moth species, others produce sound as a courtship song^12^ or for territory defence^13^. Several moth taxa produce sound by stridulation^5,14–16^, however, the majority do so with tymbals^17^: thin areas of cuticle, almost exclusively on the body, backed by an air cavity, which are buckled by a dedicated muscle. It is this buckling that produces sounds, generally ultrasonic clicks^18^. Alternative sound producing structures in moths are modified genital structures^5,14,19^, percussive ‘castanets’^20,21^, and tymbals placed in the tegula^22^ or forewing^23^.

**Figure 1:**
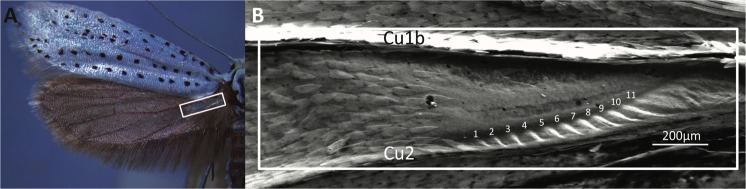
Hyaline (translucent) patch of *Yponomeuta* (**A**) *Yponomeuta evonymella* exposing the hyaline patch on its hindwing (in white box; see **B**). (**B**) Scanning electron micrograph (SEM) of the ventral side of the hyaline patch (white box corresponds to that in **A**), with the striations numbered from left to right and the Cu_1b_ and Cu_2_ veins labelled.

*Yponomeuta* Latreille, [1796] (Lepidoptera; Yponomeutidae) is a genus of likely over 100 species (Agassiz, D. Personal Communication, Mar 2018) of small (‘microlepidoptera’) to medium sized, mostly nocturnal moths^24,25^, characterised by the presence of a translucent patch devoid of scales at the hindwing base between Cu_1b_ and Cu_2_ veins (Fig. 1)^26^. Such a patch is also known from related genera of the subfamily Yponomeutinae, *Teinoptila*, *Ptiloteina*, *Trisophista*, *Eumonopyta*^27,28^. Agassiz^27^ found that these translucent patches contain a row of ridges adjacent to the Cu_2_ vein (Fig. 1B), and proposed sound production as their function by stridulation, naming the structure itself a *stridularium*. Very little is known about the evolutionary (acoustic) arms race between bats and the ‘microlepidoptera’, let alone *Yponomeuta* specifically^29,30^, though one observation exists of *Yponomeuta evonymella* and *Y. padella* producing ultrasound during flight in the field^31^.

In this study we investigate the acoustic ecology of some Yponomeutinae and address the following questions: firstly, is the translucent patch a sound producing structure, and if so, what are the acoustic properties of the sounds it produces? Secondly, if the translucent patch is a sound producing structure, how does it function? And finally, what is the adaptive value of any sound produced by these moths, in particular with respect to the acoustic arms race with bats as auditory specialist predators?

## Results

### *Yponomeuta* produce ultrasonic clicks in flight using their translucent patches

**Figure 2:**
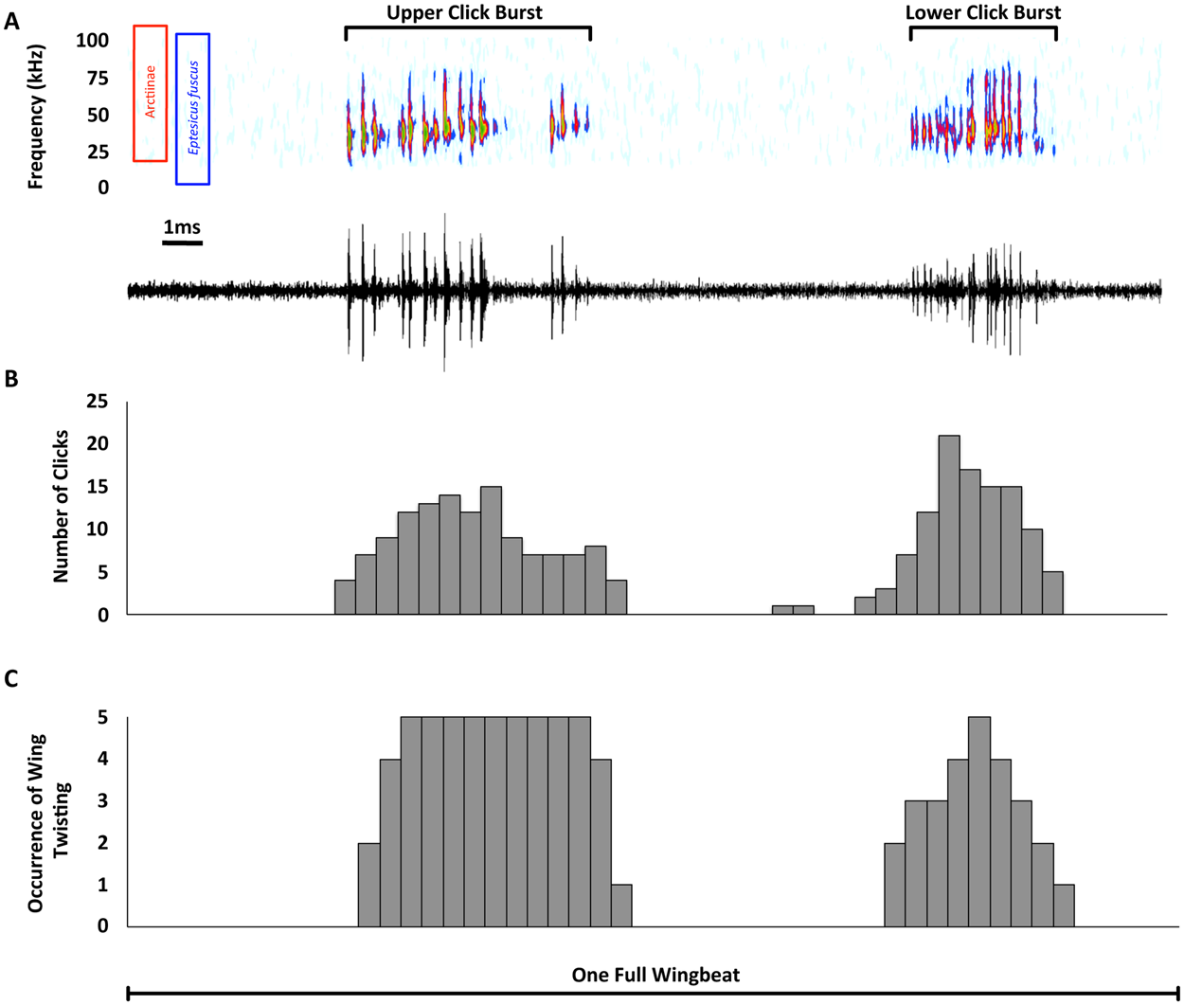
Synchronisation of click bursts with wing beats in *Yponomeuta evonymella*. (**A**) Spectrogram (FFT size 256, window Hamming, overlap 25%) and waveform showing an example of the two bursts of ultrasonic clicks produced during one wingbeat. Red and blue boxes represent the frequency range of arctiine anti-bat sounds^4^ and the hearing range of *Eptesicus fuscus* (an insectivorous bat)^36^ respectively. (**B**) Histogram of click timing over five consecutive wingbeats from one individual, time bins each show click counts for 2% of a full wingbeat cycle. (**C**) Histogram of the timing of twisting at the wing joint for the same wingbeats and bin size as panel B, a value of 1 was assigned for the occurrence and 0 for absence of twisting. All plots represent one full wingbeat.

We recorded *Yponomeuta evonymella* and *Y. cagnagella* in free and tethered flight. All tested individuals (15 tethered and two free flying *Y. evonymella* and nine tethered *Y. cagnagella*) produced two bursts of a similar number of broadband ultrasonic clicks for every wingbeat cycle (Fig. 2). One burst was produced at the beginning of the upstroke (lower burst) and the other at its end (upper burst), with the clicks emitted in a more rapid succession during the former. The number of clicks per burst appears to be similar to the number of striations on the translucent patch. In *Y. evonymella* the mean number of clicks per burst was 12.6 ± 1.7 (mean ± SD, n = 14) and the number of striations was 11 + (Fig. 1B). Note however that these recordings are a superposition of the click bursts created by the two hindwings, as proven by almost identical sounds recorded with microphones on either side of the moth.

We removed both tymbals (area 260 × 800 µm; see Fig. 1) in 12 tethered *Y. evonymella*, recorded their flight sounds pre- and post-ablation, and determined the number of clicks produced per 100 ms (about three wingbeat cycles) as this is the duration used in the literature for calculating parameters such as maximum duty cycle^4^. Post-ablation, seven individuals produced no clicks, the eighth individual produced one click, and the remaining four produced fewer clicks with lower amplitudes. Microscopic examination showed that in these four individuals ablation of the translucent patch had been incomplete, so these were excluded from further analysis. A paired-samples t-test revealed a highly significant difference (n = 8, *t*(7) = 20.3, *p* < 0.001) between the two treatments.

### *Yponomeuta* do not respond to ultrasound

Twenty *Y. evonymella* and four *Y. cagnagella* were used in hearing experiments. While in flight, no individual of either species reacted to the playback of an ultrasonic pulse known to elicit reactions of moths possessing ultrasonic hearing^32^. There was no flight cessation, or even alteration in flight direction. The 20 *Y. evonymella* individuals were exposed to the stimulus while resting as a group within a flight cage, as were the four *Y. cagnagella*, in a separate cage. None of these resting individuals showed any response, such as twitching, movement cessation, or flight initiation to ultrasound playback. Ten *Y. evonymella* were also left in a flight cage and their responses to each other observed. As with the playbacks, no individuals showed any change in resting behaviour in response to take-off or flight, and therefore sound production, of any other moth.

### Sound production does not involve stridulation but wing motion

High-speed infrared videos of *Y. evonymella* and *Y. cagnagella* in tethered flight revealed that there was no contact of any body part (potentially serving as scraper) with the translucent patch during sound production or any other phase of the wingbeat cycle. So *Yponomeuta* do not produce sound by stridulation. Instead, clicks exclusively and always occur while the hindwing rotates (pronates or supinates) along its base-to-tip axis during the upper and lower turning phases of a wing stroke (Fig. 2 and see Supplementary Video S1). More detailed analysis showed that during supination at the beginning of the upstroke the posterior anal and jugal areas of the hindwing fold downwards relative to its anterior remigium, along what is likely the claval furrow.

This folding progresses from the tip to the base of the wing including the translucent patch, and its folding coincides with the production of the lower click burst (see Supplementary Video S2). During pronation at the top of the upstroke the upper click burst is produced, but no equally obvious folding of the hindwing occurs.

### Duration, spectrum, source level, directionality, detection distance and duty cycle of clicks

Ten clicks recorded laterally (90°) from each of 14 *Y. evonymella* and nine *Y. cagnagella* were analysed for duration, temporal, amplitude and spectral parameters (Table 1). To measure horizontal emission directionality eight individuals (only six for 45°) were recorded from 0°, 45°, 90°, and 180° and five clicks analysed each (n = 150).

**Table 1:** Acoustic properties (mean ± SD; n = sample size) of the clicks of *Yponomeuta evonymella* (14 individuals) and *Y. cagnagella* (nine individuals) from 10 consecutive wingbeat cycles. Peak, low and high (highest and lowest frequency 15 dB below the amplitude of the peak frequency) frequencies were measured from spectra (Hamming window size 1024). Peak-equivalent source levels at 0.1 m were measured from waveforms and turned into detection ranges using an adaptation of the sonar equation.

Species	*Y. evonymella*	*Y. cagnagella*
Source Level (dB peSPL 0.1 m)	57.5 ± 2.6 (n = 140)	64.5 ± 0.6 (n = 90)
Peak Frequency (kHz)	37.8 ± 5.6 (n = 140)	43.5 ± 3.9 (n = 90)
Low Frequency (kHz)	23.1 ± 2.52 (n = 140)	21.2 ± 1.8 (n = 90)
High Frequency (kHz)	67.9 ± 9.72 (n = 140)	97.1 ± 2.9 (n = 90)
Click Detection Distance (m)	8.1 ± 0.6 (n = 140)	10.5 ± 0.8 (n = 90)
Click Duration (μs)	26.3 ± 3.3 (n = 280)	28.9 ± 3.6 (n = 180)
Lower Burst Click Duration (μs)	27.4 ± 2.5 (n = 140)	26.6 ± 6.7 (n = 90)
Upper Burst Click Duration (μs)	25.2 ± 3.3 (n = 140)	31.1 ± 5.0 (n = 90)
Duty Cycle (%)	1.9 ± 0.4 (n = 280)	3.4 ± 0.8 (n = 180)
Number of Clicks per Burst	11.9 ± 1.1 (n = 280)	18.8 ± 4.1 (n = 180)

**Figure 3:**
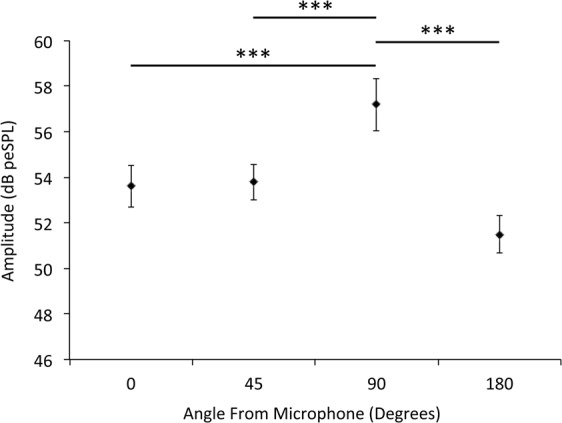
Source level directionality of *Yponomeuta evonymella* clicks Mean source level (in dB peSPL re 0.1 m) of eight *Yponomeuta evonymella*, recorded from four different directions, 0° corresponds to the microphone positioned anteriorly to the moth, 180° posteriorly, 90° laterally, and 45° anterio-laterally. Asterisks indicate significant differences of a nested ANOVA using a mixed effect model with a Bonferroni-corrected Tukey post-hoc test. Error bars show standard deviation.

**Figure 4:**
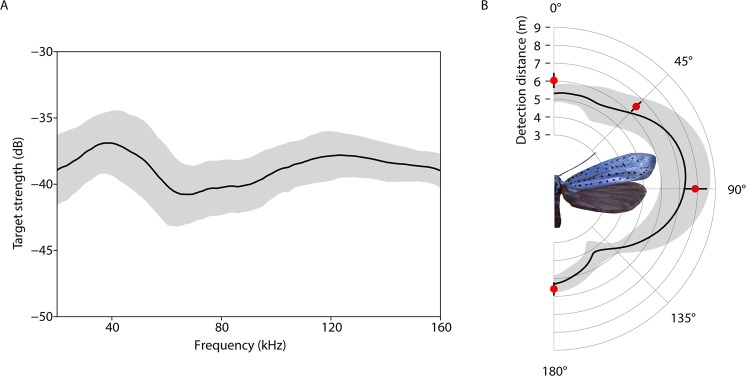
Echo strength and detection distances (**A**) Spectral target strength (black line: mean, grey area: SD, n = 5) of five dried *Y. evonymella* with the wings in an upright position to represent a mid-flight moth. (**B**) Directionality of detection distance of the right-hand side of *Yponomeuta evonymella* based on echo target strengths over 180° in 0.5° steps (black line: mean, grey area: SD, n = 5), and sound produced by the moth’s wing-based tymbal during tethered flight in four orientations; 0°, 45°, 90°, and 180° (red circles: mean, error bars SD, n = 8, 80). Photograph (c) L. O’Reilly.

In terms of the horizontal directionality of *Y. evonymella* clicks, pairwise comparisons following a nested ANOVA (n = 264, F(1,3) = 7145.475, p < 0.001) showed that the sounds recorded laterally were significantly louder than those recorded at the three other angles (0° and 90°, Z = 6.6, p < 0.001, 45° and 90°, Z = 5.8, p < 0.001, and 180° and 90°, Z = −9.0, p < 0.001) (Fig. 3). There were no differences between the other three orientations (0° and 45°, Z = 0.3, p = 1.0, 0° and 180°, Z = −2.3, p = 0.13, 45° and 180°, Z = −2.4, p = 0.11) (Fig. 3). Mean calculated distances over which bats can detect these clicks were 6.0 ± 0.4 m (n = 8, 40) at 0°, 6.5 ± 0.4 m (n = 6, 30) at 45°, 7.9 ± 0.7 m at 90°, and 5.6 ± 0.4 m at 180° (n = 8, 40) (Fig. 4B).

### Echo detection distances of *Yponomeuta* by bat biosonar

Echoes of five *Y. evonymella* were measured to determine over what distances they would be detectable to the biosonar of insectivorous bats. Spectral target strength (the signal amplitude reflected back to the receiver compared to the incident amplitude at each frequency) ranged between −35 and −43 dB at all frequencies between 20 and 160 kHz (Fig. 4A). Total target strength was highest when the moth was at 90° to the bat corresponding to a mean echo detection distance of 7.1 ± 1.1 m (n = 5) for frequencies between 20–30 kHz, and at its lowest when it was 177° to the bat with a mean echo detection distance of 4.3 ± 0.8 m (n = 5) at these frequencies (Fig. 4B).

## Discussion

### The translucent hindwing patches of *Yponomeuta* likely act as buckling tymbals

Several lines of evidence corroborate the translucent patch as a buckling tymbal. First, translucent patch structure is strikingly convergent to the ultrasound emitting tymbals found on the thorax of many arctiine moths^18,33^. In both, similarly sized thin areas of cuticle, with air on either side, consist of a larger smooth area (window) with a series of parallel striations of increasing length (band of microtymbals) running alongside it (Fig. 1). In arctiines, an inward muscular pull buckles the microtymbals in sequence and creates a burst of individual clicks. When the muscle relaxes, elastic forces buckle the microtymbals back in the reverse order creating a similar second burst of clicks^18,34^. The peak frequency of individual clicks increases during one burst and then decreases in the other, which is in agreement with the reverse order of buckling in and out (Fig. S4). Almost identically, *Yponomeuta* sounds also consist of two alternating click bursts with concurrent increases and decreases in individual click peak frequencies (Fig. 2A; Supplementary Audio S3, Fig. S4). Additionally, the mean number of clicks per burst (Table 1) is just above the number of striations (Fig. 1). Note though that the tymbals in the two hindwings operate in parallel, thereby theoretically creating twice as many clicks per burst than there are striations. Our recordings show that the moth body does not cast an effective sound shadow such that the clicks from both hindwings reach to either side. We propose that the observed mean number of clicks per burst is only about half the theoretical maximum for both wings combined because many clicks will coincide between sides, some neighbouring striations might buckle together, and some clicks may be too faint to be detected amongst other louder clicks. In summary, we conclude that the striations act as microtymbals, and that they are convergent in structure and mechanism to the sound production by microtymbals of arctiines. It can even be speculated that the tymbal deformations leading to microtymbal buckling might be similar.

The actuation mechanisms creating these tymbal deformations are however fundamentally different. In arctiines, direct muscle actuation deforms the tymbal, while in *Yponomeuta*, flight muscles at the base of the wing are the actuators, and the tymbal is deformed by the rotation and aeroelastic folding of the hindwing along the claval furrow (directly adjacent to the microtymbals of the translucent patch) during the wingbeat cycle. Because the actuation of the translucent patch is due to aerodynamic forces and the aeroelastic properties of the wing, we are terming it the ‘aeroelastic tymbal’. The evidence supports that the claval furrow is integral to the actuation of tymbal buckling, the exact biomechanical buckling mechanism is still unclear though.

### *Yponomeuta* acoustically mimic arctiine anti-bat warning sounds

Both species of *Yponomeuta* produced two bursts of ultrasonic clicks similar to those of the Arctiinae, with peak frequencies within both the hearing range of bats and the range of frequencies produced by arctiines, including sympatric species *Arctia caja* and *Phragmatobia fuliginosa* (16.6 to 109.5 kHz; Fig. 2A)^4,35,36^. The sounds even show similar increases and decreases in frequency associated with the two bursts (Fig. 2A, Supplementary Audio S3, Fig. S4). In conjunction with the lack of hearing, and therefore the lack of acoustic intraspecific communication, this suggests an anti-bat function. However, the remarkable acoustic difference to arctiines is that *Yponomeuta* sounds are produced constantly during flight. All other Lepidoptera produce sound only at specific times, for example during courtship, territory defence, or in response to the perceived presence of bats^1,21,37^. Perpetually casting its protective sound signal is inevitable for a deaf moth unable to detect and react to approaching bats.

*Yponomeuta* sounds appear to be directed at bats, but to what effect? The constant nature of *Yponomeuta* sound production eliminates the possibility of startle being the main mechanism of defence, as bats would habituate to these sounds and even use them as cues to find prey^38^. Arctiine anti-bat sounds used for aposematism/mimicry differ characteristically from those for sonar jamming in their maximum duty cycle (the percentage of time a signal is ‘on’) and the number of clicks per modulation cycle (the number of clicks per two bursts i.e. per buckling in and out of a tymbal)^4^. Whilst *Y. evonymella* and *Y. cagnagella* produce more clicks per modulation cycle than typical aposematic signalling arctiines, their duty cycles of 1.9 and 3.4% respectively place them exactly within the aposematic range. These low duty cycle anti-bat sounds are unable to jam biosonar, as a duty cycle of 20% or more is essential^1,4^. We hence conclude that *Yponomeuta* are acoustically mimicking the aposematic anti-bat sounds of tiger moths. The efficiency of these sounds as a defence should however be quantified through behavioural tests with bats.

### *Yponomeuta* employ acoustic Müllerian mimicry

The toxicity or unpalatability of an organism indicates whether their mimicking warning signals are truly aposematic (Müllerian mimicry) or impostors (Batesian mimicry). *Yponomeuta* larvae tend to be monophagous or at least limited to only a few species of food plant, something often associated with Lepidoptera that sequester specific toxins^39^. Principally, their hosts tend to be from Celastraceae, Rosaceae, Salicaceae and Crassulaceae^40^. Celastraceae and Crassulaceae contain butenolides^41^, secondary metabolites the derivatives of which are reported to have cytotoxic activity^42^, and Salicaceae contain salicin, a secondary metabolite known to act as a deterrent to insects and mammals^40,43,44^. *Yponomeuta cagnagella* larvae feed on *Euonymus europaeus* (European spindle tree; Celastraceae) which contains two butenolides, siphonodin and to a lesser extent isosiphonodin^41^. Isosiphonodin is found in *Y. cagnagella* and is either synthesised or sequestered by the insect^41^. Interestingly, isosiphonodin is also found in adults of *Yponomeuta* species that do not feed on butenolide-containing plants^41^, providing evidence that at least these species synthesise butenolides. Presence of Isosiphonodin in several species of *Yponomeuta* suggests that the compound is important in the ecology of these insects. Additionally, *Prunus padus* (Bird cherry, Rosaceae), the food plant of *Y. evonymella*, contains glucosides that can release hydrogen cyanide upon digestion, which has led to cattle poisoning^45^.

Unpalatability to predators is an obvious proposal for a function of containing butenolides and other noxious compounds. In fact, birds became drowsy when force-fed *Yponomeuta* adults^40^. However, as Menken *et al*.^40^ observed, neither larvae nor adults of *Yponomeuta* are obviously visually aposematic. We show that they use acoustic aposematism instead. These moths produce sounds with properties extremely similar to the aposematic signals of larger moths (particularly arctiines), and are mostly nocturnal and therefore at low risk of predation by birds, explaining the lack of visual aposematism. Similarly, highly nocturnal Arctiinae tend to be acoustically aposematic but visually cryptic^46^. So these butenolides, and probably other compounds such as glucosides and salicin, are likely a defence against bats. We believe that *Yponomeuta* sounds are warning bats of the presence of distasteful and potentially toxic compounds in these moths. Thus, at least the species of *Yponomeuta* containing such compounds are aposematic signallers and therefore Müllerian not Batesian mimics of arctiines.

### *Yponomeuta* sounds do not increase their conspicuousness to hunting bats

The continuous nature of *Yponomeuta* sound production might render them more vulnerable to bats because they will be able to eavesdrop on and be attracted to the warning sounds. So reduced click amplitude might be adaptive, and *Yponomeuta evonymella* clicks are indeed on average around 22 dB fainter than those of arctiines^4^. On the other hand, too low an amplitude and bats might detect the warnings too late to avoid fatal interactions. Therefore, the most adaptive warning click would be perceivable over the exact distance that a bat would detect the insect’s echoes anyway, and this is what we found for all orientations we tested (mean differences of 0.7, 0.5, 0.6 and 0.3 m for 0°, 45°, 90°, and 180° respectively; see Fig. 4B). *Yponomeuta*’s zone of acoustic protection has evolved to be just large enough to cover their zone of detectability by echolocation.

In conclusion, the aeroelastic tymbal of *Yponomeuta* is a completely novel sound-producing structure in Lepidoptera. Whilst wing-based sound production exists within the order^13,23,47^, all are evolutionarily independent of the aeroelastic tymbal and none are used to produce anti-bat ultrasound. This tymbal is a striking example of both structural and acoustic convergent evolution in the bat-moth evolutionary arms race, as well as being remarkable as a passive acoustic defence mechanism that bypasses the need for predator detection. The use of acoustic Müllerian mimicry by a deaf moth in the bat-moth evolutionary arms race shows again how little we know of the complex acoustic war raging in the night skies.

## Materials and Methods

### Specimens

Live specimens of two British *Yponomeuta* species (Yponomeutidae, Lepidoptera), *Y. evonymella* and *Y. cagnagella* were used during the investigation. All specimens were wild caught as larvae and reared to pupation by donors. Pupae were kept in the laboratory until eclosion within 297 × 159 × 102 mm plastic rearing boxes (WorldwideButterflies, Lulworth, United Kingdom) at 21 °C. Due to initially having much higher numbers of the former than the latter, ablation and directionality experiments were performed using *Y. evonymella* only while sound production was documented and analysed in both species. As numbers of available individuals were limited, all individuals were flown until they became exhausted and would no longer fly.

### Tethering

Moths were tethered for most high-speed video recordings as well as all ablation experiments. Due to their small size, standard methods of tethering such as adhesives failed, so we inserted a size 000 insect pin dorsally into the mesothorax/prothorax of the moth until it just protruded ventrally. The pinhead was attached to a piece of dowel (5 mm in diameter) which itself was clamped so the moth was suspended in the centre of the flight arena. Although this is obviously an invasive tethering method, tethered specimens continued to fly for prolonged periods of time. Both audio and video recordings showed no obvious difference in the sounds produced by moths or their behaviour between tethered and free flight, so we continued with this as our tethering method.

### Sound playback

Twenty *Y. evonymella*, and four *Y. cagnagella* were free flown in a semi-anechoic chamber with and without exposure to an ultrasonic stimulus. Two human observers documented the behaviour of each individual under both conditions. A reaction was defined as any typical anti-bat escape manoeuvre including sudden cessation of flight or change in flight direction. Each moth was flown twice and one observer chose the order of stimulus exposure for each individual, while the other observer was kept blind to the stimulus condition. A Dazer II Ultrasonic Dog Deterrent (Dazer International, London, UK) was used as the stimulus, between one and two metres from the subject. The Dazer II produces a 25 kHz tone at 118.1 dB SPL (at 0.1 m). All 24 individuals (separated by species) were also ensonified at rest within a 24 × 24 × 24″ BugDorm-1 Insect Rearing Cage (Megaview Science Co., Ltd., Taichung City, Taiwan), at a distance of around 1 m from the centre of the cage.

### Audio recordings

All recordings were made using a Type 4954 ¼″ free-field microphone (grid on) with a Type 2669-L preamplifier, connected to a Type 2690 NEXUS conditioning amplifier (all Brüel & Kjær Sound and Vibration Measurements A/S, Nærum, Denmark), run through National Instruments NI-USB-6251 BNC sound card (National Instruments, Austin, Texas, United States).

For audio and high-speed video recordings insects were released or tethered within a 24 × 24 × 24″ BugDorm-1 Insect Rearing Cage (Megaview Science Co., Ltd., Taichung City, Taiwan) lined on the base, back and one wall with ultrasound absorbing foam (Studiofoam 4″ Pyramids, Auralex Acoustics Inc., Indianapolis, IN) to reduce echoes and reverberation. The recording microphone was positioned through a small circular hole cut into the mesh on the unlined side of the cage. The front panel (facing the camera) and the right-hand panel of the BugDorm-1 were removed for synchronous audio and video recordings in order to facilitate the activation of the synchronisation click.

The software used to make the recordings was AviSoft Ni-Daq Recorder (Avisoft Bioacoustics, Berlin, Germany). All audio recordings were 16 bit, recorded at a sampling rate of 300 kHz, with the microphone between 7 and 13.5 cm from the moth for tethered flight.

### High-speed video recordings

Video recordings were made in the same set-up as above with the camera (Photron FASTCAM SA1.1, Phtoron, Tokyo, Japan) lens (Nikon Micro-NIKKOR 105 mm prime lens, Nikon, Tokyo, Japan) positioned through a sleeve opening of the BugDorm-1 and pointing perpendicular to the microphone axis. Video recordings were made at 3000 fps with a resolution of 1024 × 1024 pixels and the subject was illuminated using infrared (IR, 850 nm) lighting from four LED light sources.

Video and audio recordings were synchronised with the use of a pair of pliers. The pliers were kept in frame and when closed they produced an extremely short broadband click which allowed for very accurate synchronisation of frame (video) and sample (audio) number. Synchronisation frames and samples were those that contained the collision of the jaws of the pliers and the beginning of the click respectively.

### Ablation

Twelve *Y. evonymella* were tethered, flown, and recorded with their hindwings intact. The moths were positioned in the flight set-up and left, holding a small piece of foam to simulate being sat on a surface for 15–30 minutes or until they initiated flight themselves. If they had not initiated flight by then, it was elicited by removing the piece of foam they were holding, which reliably triggered flight.

Under a 50x magnification dissection microscope (Leica EZ5 Stereo Microscope, Leica Microsystems, Wetzlar, Germany) the translucent patches in both hindwings were then removed using microdissection scissors from the wing joint to the point where scales began to appear. All ablated individuals were alive after that treatment and continued to fly on a tether with no noticeable difference in their flight pattern and readiness. Their sounds were then recorded again using the same procedure.

### Sound Emission Directionality

The directionality of the click amplitude of eight, tethered Y. evonymella was quantified by recording the sounds using the same setup with the microphone facing the moth from four orientations with respect to the longitudinal axis of the moths from distances between 11.5 and 13.3 cm. Moth sounds were recorded from anterior (microphone at 0° facing the moth), posterior (microphone at 180°, behind the moth), lateral (at 90° from the right side of the moth) and anterio-lateral (microphone at 45° to the moth’s right side) directions. For each individual at each orientation the loudest click was isolated from the upper click burst for five consecutive wingbeats. Full bursts of clicks could not be detected from anterio-lateral recordings from two individuals and so the number of clicks at this orientation was 30 from six individuals as opposed to 40 from eight for the other orientations.

### Acoustic analysis

All sound recordings were analysed using Avisoft SASLab Pro (version 5.2.07, Avisoft Bioacoustics, Berlin, Germany). For each individual, click bursts from ten consecutive wingbeats were analysed, either counting all clicks or further analysing the loudest click from each upper click burst. Click number was determined by totalling the number of clicks discernible in waveform and spectrogram for each of the two click bursts. Individual click duration was measured manually from the waveform. Click amplitude was calculated as peak-to-peak sound pressure values using the waveform of individual clicks, and was then converted to dB peSPL using a calibrated 40 kHz signal generator (Avisoft Bioacoustics, Berlin, Germany) and using the following formula:

$$CA+20\ast lo{g}_{10}(\frac{TS}{CS})$$

$$CA={Calibration}\,{Tone}\,{Amplitude}\,({dB})$$

$$TS={Test}\,{Signal}\,{Pressure}\,({\rm{Pa}})$$

$$CS={Calibration}\,{Signal}\,{Sound}\,{Pressure}\,({\rm{Pa}})$$.

For spectral analysis, individual clicks were isolated from the waveform including a linear ramp of 0.05 ms of noise on either side. Silence was then added (zero padding) on either side before analysis. Peak frequency was determined from a power spectrum (Hamming window size 1024). High and low frequency values are the frequencies ± 15 dB below the amplitude of the peak frequency.

### Calculation of detection distance of *Yponomeuta* sounds

Click detection distances were calculated for 14 *Yponomeuta evonymella* individuals. For each individual the loudest click from the upper burst of ten consecutive wingbeats was analysed. The peak frequencies and source level (dB peSPL) of each click were used to calculate the distance at which these sounds could be detected by bats, using a hearing threshold of 10 dB SPL. The following formula, an adaptation of the sonar equation^48^, was used to calculate these distances.

$$CSL-20\ast lo{g}_{10}(\frac{\delta -\delta ref}{\delta ref})-FDA\ast (\delta -\delta ref)=HT$$

$$HT={\rm{hearing}}\,{\rm{threshold}}=10\,{\rm{dB}}\,{\rm{SPL}}$$

$$CSL={\rm{Click}}\,{\rm{Source}}\,{\rm{Level}}\,({\rm{dB}}\,{\rm{peSPL}}\,{\rm{at}}\,{\rm{\delta }}\mathrm{ref})$$

$$\delta =\mathrm{Distance}\,({\rm{m}})$$

$$\delta ref={\rm{Reference}}\,{\rm{Distance}}=0.1\,{\rm{m}}$$

$$FDA={\rm{Frequency}}\,{\rm{Dependent}}\,{\rm{Attenuation}}\,(\mathrm{dB}\,{{\rm{m}}}^{-1}){\rm{.}}$$

*Yponomeuta evonymella* directional click detection distance was calculated in the same manner, but using five consecutive wingbeats per angle (0°, 45°, 90°, and 180°) from eight individuals.

### Moth echo detection distances

Echo strength of whole insect specimens was measured as spectral target strength (the fraction of the impinging sound energy returned from the target). Live specimens were killed by freezing and set with their wings in an upwards direction with the leading edge of the forewing perpendicular to the longitudinal axis of the body. The target specimen was positioned on a vertical tower (27.8 cm high, 2.5 × 5 cm wide) of ultrasound absorbing foam (Basotect W, BASF, Ludwigshafen, Germany) placed on a turntable (LT360, LinearX Systems Inc., Battle Ground, WA). A sonar measurement head mounted on a lever arm faced the target from a lateral distance of 31 cm. The sonar measurement head consisted of a ¼″ ultrasound microphone (type 26AB without protective grid), pre-amplifier (type 2669 L, both GRAS Sound & Vibration A/S, Holte, Denmark), microphone power supply (type 5935-L, Brüel & Kjær, Nærum, Denmark) and a custom-made ferro-electret foil loudspeaker (33 × 14 mm, Emfit Ltd., Vaajakoski, Finland) powered by a PZD350 M/S high-voltage amplifier (TREK Inc., Lockport, NY). The centres of the microphone and speaker were separated by 15 mm, roughly replicating the distance between the mouth and ears of a bat. The turntable, speaker, and microphone were connected to a soundcard (NI-DAQ BNC-2110) controlled using custom-programmes (LabVIEW v.16.0; both National Instruments, Austin, TX). For detailed methods see^49,50^. Five individual *Yponomeuta evonymella* were analysed using this technique. Each moth was scanned from 0–180° in 0.5° steps in the horizontal plane. A frequency modulated sweep from 15 to 250 kHz was used to ensonify the moth and for each position four echoes were recorded and averaged.

Detection distances of moth echoes were calculated analogous to click detection distances, but for two-way spherical transmission losses and with FDA for bat call frequencies with the highest detection range in the UK, i.e. at 20–30 kHz:

$$BSL-2\ast 20\ast lo{g}_{10}(\frac{\delta -\delta ref}{\delta ref})-2\ast FDA\ast (\delta -\delta ref)+TS=HT$$

$$TS={\rm{spectral}}\,{\rm{target}}\,\mathrm{strength}\,\,{\rm{of}}\,{\rm{moth}}\,{\rm{echo}}\,({\rm{dB}}\,{\rm{at}}\,\delta ref)$$

$$BSL={\rm{Source}}\,{\rm{Level}}\,{\rm{of}}\,{\rm{bat}}\,{\rm{call}}\,({\rm{dB}}\,{\rm{peSPL}}\,{\rm{at}}\,\delta ref)$$

### Statistics

All statistical tests were performed using R studio (R version 3.1.2.). A two-tailed paired samples t-test was performed to compare the number of clicks produced before and after ablation of the aeroelastic tymbals. A two-tailed nested ANOVA run as a mixed effects model, with moth individual as the random effect, was used to test for differences between the amplitudes of *Yponomeuta* sounds recorded at different angles. Moth individual was nested within the angle at which it was recorded. This was followed by a Tukey post-hoc test with Bonferroni correction.

## Supplementary information

Supplementary figure S4

Supplementary Video S1

Supplementary Video S2

Supplementary Audio S3
