# Supplementary material for: Deaf moths employ acoustic Müllerian mimicry against bats using wingbeat-powered tymbals
Source: Sci Rep. 2019 Feb 5;9:1444. doi: 10.1038/s41598-018-37812-z (PMC6363749; doi:10.1038/s41598-018-37812-z)
Supplement: Supplementary file 1 — Supplementary figure S4 [file 41598_2018_37812_MOESM1_ESM.docx]

## Supplementary Material

# for

# Deaf moths employ acoustic Müllerian mimicry against bats using wingbeat-powered tymbals

**Liam J. O’Reilly^1^, David J. L. Agassiz ^2^, Thomas R. Neil^1^, Marc W. Holderied^1^**

1 School of Biological Sciences, University of Bristol, Bristol, UK

2 Department of Life Sciences, Insect Division, Natural History Museum, London, UK

Corresponding Author – Marc Holderied (marc.holderied@bristol.ac.uk)

**Supplementary Video S1.** High-speed (3000fps) video and synchronised audio of *Yponomeuta cagnagella* in flight, slowed down 100 times. Folding of the aeroelastic tymbal along the claval furrow can clearly been seen during at the lower phase of the wingbeat, and this folding coincides exactly with sound production, which can be visualised using the real-time spectrogram on the right-hand side of the video. Areas of the wing are anatomically labelled to aid with interpretation.

**Supplementary Video S2.** High-speed (3000fps) video and synchronised audio of *Yponomeuta cagnagella* in flight, slowed down 100 times. Twisting of the hindwing joint can be seen at the top of the wingbeat, as can folding of the hindwing at the bottom of the wingbeat, both events coincide with sound production which can be visualised using the real-time spectrogram on the right-hand side of the video. Areas of the wing are anatomically labelled to aid with interpretation.

**Supplementary Audio S3.** Audio recording of *Yponomeuta cagnagella* showing increases and decreases in pitch within the alternating click bursts. The recording has been slowed down thirty times. Comparisons can be made to the Supplementary material of Corcoran & Hristov (2014) (DOI https://doi.org/10.1007/s00359-014-0924-0) ^1^which shows the same pattern of pitch change in the aposematic signals of *Cycnia tenera* (Arctiinae, Erebidae), and its mimic *Eubaphe unicolor* (Geometridae).


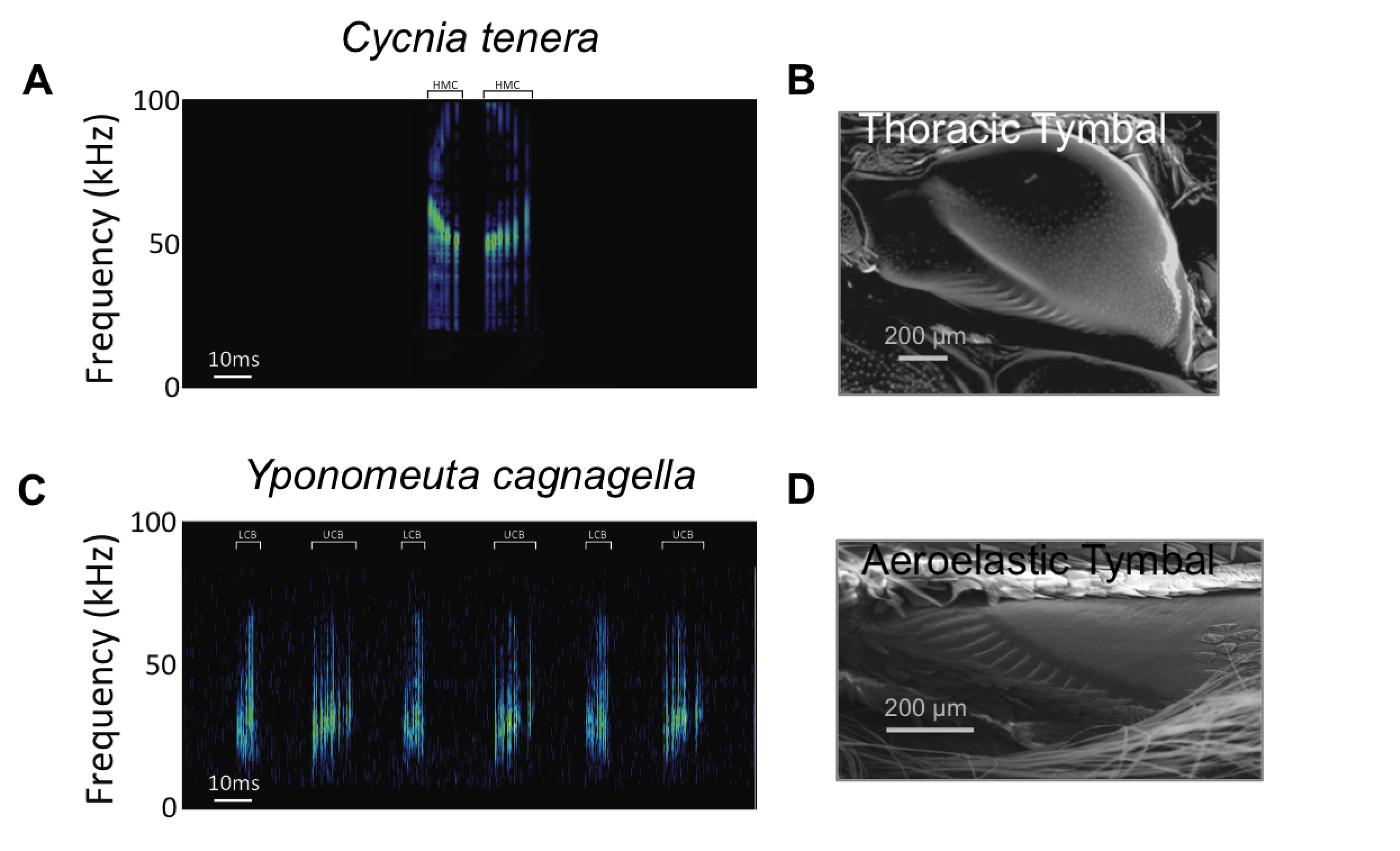


**Supplementary Figure S4.** (A) Spectrogram of *Cycnia tenera* (Arctiinae) demonstrating the decrease and then increase in pitch of the two half modulation cycles (HMC) or click bursts (B) SEM of the thoracic tymbal of *C. tenera*. Both panels (A) and (B) are modified with permission from Barber et al. (2007) (Copyright (2007) National Academy of Sciences, U.S.A.) ^2^.(C) Spectrogram (FFT 256, window Hamming, overlap 25%, not calibrated for amplitude) of sounds produced during three full wingbeats of *Yponomeuta cagnagella* showing the decreasing pitch of the click burst produced during the lower phase (Lower Click Burst, LCB) of the wingbeat and the subsequent increasing pitch of the click burst produced during the upper phase (Upper Click Burst, UCB). (D) SEM of aeroelastic tymbal of *Y. cagnagella*.

# References

1. Corcoran, A. J. & Hristov, N. I. Convergent evolution of anti-bat sounds. *J. Comp. Physiol. A* **200,** 811–821 (2014).

2. Barber, J. R. & Conner, W. E. Acoustic mimicry in a predator-prey interaction. *Proc. Natl. Acad. Sci. U. S. A.* **104,** 9331–9334 (2007).
